# Distribution of cardiovascular disease and retinopathy in patients with type 2 diabetes according to different classification systems for chronic kidney disease: a cross-sectional analysis of the renal insufficiency and cardiovascular events (RIACE) Italian multicenter study

**DOI:** 10.1186/1475-2840-13-59

**Published:** 2014-03-13

**Authors:** Giuseppe Pugliese, Anna Solini, Enzo Bonora, Emanuela Orsi, Gianpaolo Zerbini, Cecilia Fondelli, Gabriella Gruden, Franco Cavalot, Olga Lamacchia, Roberto Trevisan, Monica Vedovato, Giuseppe Penno

**Affiliations:** 1 Department of Clinical and Molecular Medicine, “La Sapienza” University, Via di Grottarossa, 1035-1039, 00189 Rome, Italy; 2 Department of Clinical and Experimental Medicine, University of Pisa, Pisa, Italy; 3 Division of Endocrinology and Metabolic Diseases, University of Verona, Verona, Italy; 4 Endocrinology and Diabetes Unit, Department of Medical Sciences, Fondazione IRCCS “Cà Granda – Ospedale Maggiore Policlinico”, Milan, Italy; 5 Complications of Diabetes Unit, Division of Metabolic and Cardiovascular Sciences, San Raffaele Scientific Institute, Milan, Italy; 6 Diabetes Unit, Department of Internal Medicine, Endocrine and Metabolic Sciences and Biochemistry, University of Siena, Siena, Italy; 7 Department of Internal Medicine, University of Turin, Turin, Italy; 8 Unit of Internal Medicine, Department of Clinical and Biological Sciences, University of Turin, Turin, Orbassano, Italy; 9 Unit of Endocrinology and Metabolic Diseases, Department of Medical Sciences, University of Foggia, Foggia, Italy; 10 Diabetes Unit, Hospital of Bergamo, Bergamo, Italy; 11 Department of Clinical and Experimental Medicine, University of Padua, Padua, Italy

**Keywords:** Chronic kidney disease, Classification, eGFR, Albuminuria, Cardiovascular disease, Diabetic retinopathy

## Abstract

**Background:**

The National Kidney Foundation’s Kidney Disease Outcomes Quality Initiative (NKF’s KDOQI) staging system for chronic kidney disease (CKD) is based primarily on estimated GFR (eGFR). This study aimed at assessing whether reclassification of subjects with type 2 diabetes using two recent classifications based on both eGFR and albuminuria, the Alberta Kidney Disease Network (AKDN) and the Kidney Disease: Improving Global Outcomes (KDIGO), provides a better definition of burden from cardiovascular disease (CVD) and diabetic retinopathy (DR) than the NKF’s KDOQI classification.

**Methods:**

This is a cross-sectional analysis of patients with type 2 diabetes (n = 15,773) from the Renal Insufficiency And Cardiovascular Events Italian Multicenter Study, consecutively visiting 19 Diabetes Clinics throughout Italy in years 2007-2008. Exclusion criteria were dialysis or renal transplantation. CKD was defined based on eGFR, as calculated from serum creatinine by the simplified Modification of Diet in Renal Disease Study equation, and albuminuria, as measured by immunonephelometry or immunoturbidimetry. DR was assessed by dilated fundoscopy. Prevalent CVD, total and by vascular bed, was assessed from medical history by recording previous documented major acute events.

**Results:**

Though prevalence of complications increased with increasing CKD severity with all three classifications, it differed significantly between NKF’s KDOQI stages and AKDN or KDIGO risk categories. The AKDN and KDIGO systems resulted in appropriate reclassification of uncomplicated patients in the lowest risk categories and a more graded independent association with CVD and DR than the NKF’s KDOQI classification. However, CVD, but not DR prevalence was higher in the lowest risk categories of the new classifications than in the lowest stages of the NKF’s KDOQI, due to the inclusion of subjects with reduced eGFR without albuminuria. CVD prevalence differed also among eGFR and albuminuria categories grouped into AKDN and KDIGO risk category 1 and moderate, respectively, and to a lesser extent into higher risk categories.

**Conclusions:**

Though the new systems perform better than the NKF’s KDOQI in grading complications and identifying diabetic subjects without complications, they might underestimate CVD burden in patients assigned to lower risk categories and should be tested in large prospective studies.

**Trial registration:**

ClinicalTrials.gov; NCT00715481

## Background

In 2002, the National Kidney Foundation’s Kidney Disease Outcomes Quality Initiative (NKF’s KDOQI) has introduced a staging system for chronic kidney disease (CKD) [1]. Though widely used for both epidemiological and clinical purposes, this 5-stage system has been criticized for being based primarily on glomerular filtration rate (GFR), as calculated by the use of equations which may not provide accurate estimates, especially in elderly and female individuals [2]. This may lead to erroneous categorization of otherwise healthy individuals with consequent overestimation of CKD prevalence and inappropriate referral to specialist [3]. In addition, the NKF’s KDOQI classification does not entirely meet the fundamental requirements for staging systems, which should reflect the natural history and particularly the prognosis of the disease.

That this system is often inadequate to describe the sequence of events occurring in CKD has become increasingly evident in view of the high prevalence of nonalbuminuric renal impairment, i.e. estimated GFR (eGFR) <60 ml/min/1.73 m^2^ without albuminuria, detected in samples from the general population [4,5] and also in patients with type 1 diabetes from the Diabetes Control and Complications Trial (DCCT)/Epidemiology of Diabetes Interventions and Complications study [6] and, to a higher extent, in subjects with type 2 diabetes from the United Kingdom Prospective Diabetes Study [7]. This latter finding has been confirmed in large cross-sectional analyses [5,8] and in the baseline evaluation of two intervention trials [9,10], thus implying that the majority of individuals with type 2 diabetes develop CKD from stage 3 of the NFK’s KDOQI classification. Therefore, the natural history of CKD in diabetes is now recognized to follow two different pathways, albuminuric and nonalbuminuric [11].

More importantly, the NKF’s KDOQI classification was originally designed to assign people with more severe prognoses to more advanced stages in order to select for referral only individuals at high risk for adverse outcomes [1]. However, for definition of stages 3-5, this classification does not incorporate information about the presence and severity of albuminuria, which is a powerful predictor of renal and cardiovascular disease (CVD) outcomes, independently of eGFR, both in non-diabetic and diabetic individuals [12-16]. The Kidney Disease: Improving Global Outcomes (KDIGO) in a Controversies Conference held in London in 2009 [17] recommended to retain the NKF’s KDOQI definition of CKD and to revise classification by including cause and albuminuria category in addition to GFR category, with subdivision of GFR category 3 at 45 ml/min/1.73 m^2^ (CGA classification). Recently, an alternate classification system based on both eGFR and albuminuria has been proposed and validated by the Alberta Kidney Disease Network (AKDN) in terms of adverse renal outcomes and, to a lesser extent, all-cause mortality. In this classification, eGFR and albuminuria categories with similar relative risks are grouped into risk categories 1-4 (plus risk category 0 corresponding to no CKD of the NKF’s KDOQI system) [18]. In the accompanying editorial, Levey *et al.* did not recommend substituting risk categories for eGFR and albuminuria categories or relying on risk categories alone to predict clinical outcomes [19]. However, in 2012, the KDIGO has also grouped eGFR and albuminuria categories into 3 risk categories (moderate, high and very high risk, plus low risk category also corresponding to no CKD of the NKF’s KDOQI system) to be used for guiding decisions and predicting outcomes [20]. So far, it has not been investigated which CKD classification system stratifies more accurately diabetic individuals by prevalence of CVD and other complications.

This study was aimed at assessing whether reclassification of subjects with type 2 diabetes from the Renal Insufficiency and Cardiovascular Events (RIACE) Italian Multicenter Study using the AKDN alternate system or the new KDIGO classification provides a better definition of burden from CVD and diabetic retinopathy (DR) than the NKF’s KDOQI classification.

## Methods

### Study cohort

In this cross-sectional analysis, we used the data collected at the baseline visit for the RIACE Italian Multicenter Study, an observational, prospective cohort study on the impact of eGFR on morbidity and mortality from CVD in subjects with type 2 diabetes.

The RIACE cohort consisted of 15,933 Caucasian patients with type 2 diabetes, attending consecutively 19 hospital-based Diabetes Clinics of the National Health Service throughout Italy (see The RIACE Study Group) in years 2007-2008. Exclusion criteria were dialysis or renal transplantation. The study protocol was approved by the locally appointed ethics committees. Then, quality and completeness of data were controlled and 160 patients were excluded due to implausible or missing values and the remaining 15,773 subjects were subsequently analyzed.

### Measurements

All patients underwent a structured interview to collect the following information: age, smoking status, known diabetes onset and duration, current glucose-, blood pressure (BP)- and lipid-lowering therapy, with indication of the class of drug. Weight and height were assessed and body mass index (BMI) calculated, then BP was measured with a sphygmomanometer after a 5-min rest. Hemoglobin A_1c_ (HbA_1c_) was measured by high-performance liquid chromatography using DCCT-aligned methods; triglycerides, total and HDL cholesterol were determined by standard analytical methods.

The presence of CKD was assessed by measuring albuminuria and serum creatinine. As previously reported in detail [8,21,22], albumin excretion rate was obtained from 24-hour urine collections or calculated from albumin/creatinine ratio in first-morning urine samples, in the absence of symptoms and signs of urinary tract infection or other interfering clinical conditions. Albuminuria was measured in one-to-three fresh urine samples for each patient by immunonephelometry or immunoturbidimetry and, in case of multiple measurements, the geometric mean was used for analysis. In subjects with multiple measurements (4,062 with at least two and 2,310 with three values), concordance rate between the first value and the geometric mean was >90% for all classes of albuminuria [21]. Patients were then assigned to one of the following categories of albuminuria (mg/24 hours): normoalbuminuria (<30), microalbuminuria (30-299), or macroalbuminuria (≥300). Serum (and urine) creatinine was measured by the modified Jaffe method. One to three measurements were obtained for each patients and eGFR was calculated by the four-variable Modification of Diet in Renal Disease Study equation [23], using the mean serum creatinine value in case of multiple measures, as previously reported [8,21]. Patients were then assigned to one of the following categories of eGFR (mL/min/1.73 m^2^): 1 (≥90); 2 (60-89); 3 (30-59); 4 (15-29); and 5 (<15). All measurements were undertaken from a standardized protocol across study centers.

The presence of DR was assessed by an expert ophthalmologist with dilated fundoscopy. Patients were classified into the following categories: absent DR, mild, moderate or severe non-proliferative DR (NPDR), proliferative DR (PDR), or maculopathy, according to the Global Diabetic Retinopathy Project Group [24]. For further analysis, patients with NPDR of mild or moderate degree were classified as having non-advanced DR, whereas those with severe NPDR or pre-PDR, PDR, maculopathy alone (i.e. without NPDR or PDR), or blindness were grouped into the advanced, sight-threatening DR category [25]. DR grade was assigned based on the worst eye.

Prevalent CVD was assessed from medical history by recording previous documented major acute CVD events, including myocardial infarction, stroke, foot ulcer or gangrene, amputation, coronary, carotid, and lower limb revascularization. CVD events were adjudicated based on hospital discharge records by an *ad hoc* committee in each center [26,27].

### Statistical analysis

**Figure 1:**
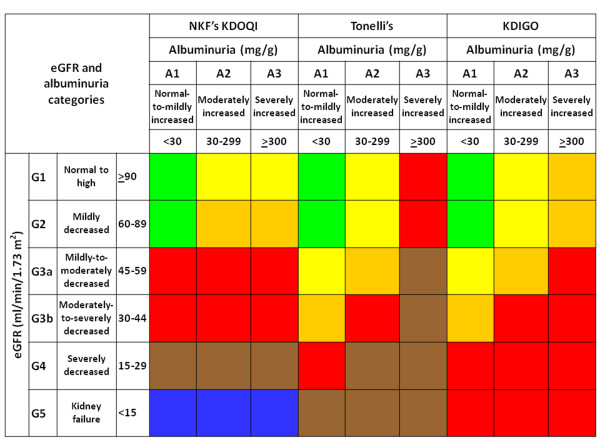
**NFK’s KDOQI, AKDN risk category, and KDIGO CKD classification systems.** NFK’s KDOQI classification: stage 0 (green), 1 (yellow), 2 (orange), 3 (red), 4 (brown), and 5 (blue); AKDN alternate system: risk category 0 (green), 1 (yellow), 2 (orange), 3 (red), and 4 (brown); KDIGO classification: risk category low (green), moderate (yellow), high (orange), and very high (red).

Based on eGFR and albuminuria levels, patients were stratified according to the NKF’s KDOQI, AKDN and KDIGO classifications (Figure 1) in no CKD and stages 1-5, risk categories 0-4, and low, moderate, high and very high risk, respectively.

Clinical data were then derived for subjects assigned to each CKD stage or risk category, together with prevalence of CVD and DR according to eGFR and albuminuria categories as well as CKD stages or risk categories. Differences among CKD stages or risk categories were evaluated using the following statistical tests: one-way ANOVA and Kruskall-Wallis for parametric and non-parametric continuous variables, respectively, and Pearson χ^2^ for categorical variables. A Mantel-Haenszel linear-by-linear association χ^2^ test for linear trend was applied for evaluating variation of prevalence values with increasing eGFR and albuminuria categories (i.e. from G0 to G5 and from A1 to A3, respectively) as well as with increasing CKD stage or risk category.

Multiple logistic regression analyses with backward variable selection (probability for removal >0.10) were performed to assess the relation of each complication with CKD stages or risk categories as well as with subcategories of AKDN risk categories 1, 2 and 3 and KDIGO risk categories moderate and high, independently of the following confounders: age, gender, smoking habits, diabetes duration, HbA_1c_, anti-hyperglycemic treatment, triglycerides, HDL cholesterol, dyslipidemia (elevated LDL cholesterol and/or specific treatment), and hypertension (elevated systolic and/or diastolic BP and/or specific treatment).

All p values were two-sided, and a p value of less than 0.05 was considered statistically significant. Statistical analyses were performed using SPSS version 13.0 (SPSS Inc., Chicago, Illinois, USA).

## Results

The clinical characteristics of subjects according to NKF’s KDOQI stages and AKDN or KDIGO risk categories are presented in Additional file 1: Tables S1-S3. Across all classification systems, age, age at diabetes diagnosis, diabetes duration, HbA_1c_, percentage of insulin users, triglycerides, non-HDL cholesterol, systolic BP, prevalence of dyslipidemia, hypertension, lipid-lowering therapy, and anti-hypertensive treatment (including usage of blockers of the renin-angiotensin system), BMI, and waist circumference tended to increase, whereas HDL cholesterol and, except in the AKDN system, percentage of current smokers decreased. Men predominated in NKF’s KDOQI stages 1-2, whereas women prevailed in stages 3-5, due to the higher percentage of females with nonalbuminuric renal impairment, the most frequent phenotype in subjects with eGFR < 60 ml/min/1.73 m^2^[5,8]. Conversely, male gender predominated in all risk categories of the AKDN and KDIGO classifications.

As previously reported [25,26], prevalence of any CVD was 23.2% (any coronary event 15.3%, acute myocardial infarction 11.2%, any cerebrovascular event 8.3%, stroke 3.3%, any peripheral event 5.7%, and ulcer/gangrene 3.4%) and that of any DR was 22.2% (non-advanced 12.4% and advanced 9.8%). Prevalence of CVD and DR increased progressively with eGFR categories (*P* for trend <0.0001), though coronary and cerebrovascular events decreased in G4-G5 and G5, respectively, likely due to a survival bias. Likewise, prevalence of all complications showed a stepwise increase from normo to macroalbuminuria (*P* for trend <0.0001) (Table 1).

**Table 1:** Cases (% of total) and number of subjects (% of cases) with any CVD, any coronary event, AMI, any cerebrovascular event, stroke, any peripheral event, ulcer/gangrene, and non-advanced and advanced DR according to eGFR and albuminuria category

**Cases**					**Any CVD**				
**N**	**A1**	**A2**	**A3**	**Total**	**n**	**A1**	**A2**	**A3**	**Total**
**G1**	3,610 (22.9)	932 (5.9)	120 (0.8)	4,662 (29.6)	**G1**	539 (14.9)	197 (21.1)	35 (29.2)	771 (16.5)
**G2**	6,255 (39.7)	1,653 (10.5)	244 (1.6)	8,152 (51.7)	**G2**	1,218 (19.5)	480 (29.0)	82 (33.6)	1,780 (21.8)
**G3a**	1,253 (7.9)	562 (3.6)	136 (0.9)	1,951 (12.4)	**G3a**	373 (29.8)	220 (39.2)	67 (49.3)	660 (33.8)
**G3b**	351 (2.2)	263 (1.7)	136 (0.9)	750 (4.8)	**G3b**	129 (36.8)	135 (51.3)	61 (44.9)	325 (43.3)
**G4**	60 (0.4)	82 (0.5)	87 (0.6)	229 (1.5)	**G4**	24 (40.0)	40 (48.8)	56 (64.4)	120 (52.4)
**G5**	9 (0.06)	5 (0.03)	15 (0.1)	29 (0.2)	**G5**	2 (22.2)	3 (60.0)	4 (26.7)	9 (31.0)
**Total**	11,538 (73.2)	3,497 (22.2)	738 (4.7)	15,773 (100.0)	**Total**	2,285 (19.8)	1,075 (30.7)	305 (41.3)	3,665 (23.2)
**Any coronary event**					**AMI**				
**N**	**A1**	**A2**	**A3**	**Total**	**n**	**A1**	**A2**	**A3**	**Total**
**G1**	369 (10.2)	122 (13.1)	17 (14.2)	508 (10.9)	**G1**	260 (7.2)	86 (9.2)	11 (9.2)	357 (7.7)
**G2**	835 (13.4)	298 (18.0)	43 (17.6)	1,176 (14.4)	**G2**	614 (9.8)	218 (13.2)	31 (12.7)	863 (10.6)
**G3a**	264 (21.1)	136 (24.2)	36 (26.5)	436 (22.4)	**G3a**	189 (15.1)	105 (18.7)	22 (16.2)	316 (16.2)
**G3b**	93 (26.5)	96 (36.5)	39 (28.7)	228 (30.4)	**G3b**	68 (19.4)	73 (27.8)	31 (22.8)	172 (22.9)
**G4**	16 (26.7)	23 (28.1)	23 (26.4)	62 (27.1)	**G4**	13 (21.7)	18 (22.0)	15 (17.2)	46 (20.1)
**G5**	1 (11.1)	1 (20.0)	3 (20.0)	5 (17.2)	**G5**	1 (11.1)	1 (20.0)	2 (13.3)	4 (13.8)
**Total**	1,578 (13.7)	676 (19.3)	161 (21.8)	2,415 (15.3)	**Total**	1,145 (9.9)	501 (14.3)	112 (15.2)	1,758 (11.2)
**Any cerebrovascular event**					**Stroke**				
**N**	**A1**	**A2**	**A3**	**Total**	**n**	**A1**	**A2**	**A3**	**Total**
**G1**	167 (4.6)	62 (6.7)	12 (10.0)	241 (5.2)	**G1**	57 (1.6)	23 (2.5)	7 (5.8)	87 (1.9)
**G2**	403 (6.4)	194 (11.7)	30 (12.3)	627 (7.7)	**G2**	169 (2.7)	80 (4.8)	13 (5.3)	262 (3.2)
**G3a**	136 (10.9)	89 (15.8)	33 (24.3)	258 (13.2)	**G3a**	52 (4.2)	28 (5.0)	13 (9.6)	93 (4.8)
**G3b**	48 (13.7)	53 (20.2)	23 (16.9)	124 (16.5)	**G3b**	20 (5.7)	22 (8.4)	8 (5.9)	50 (6.7)
**G4**	11 (18.3)	19 (23.2)	22 (25.3)	52 (22.7)	**G4**	3 (5.0)	8 (9.8)	10 (11.5)	21 (9.2)
**G5**	0 (0.0)	2 (40.0)	1 (6.7)	3 (10.3)	**G5**	0 (0.0)	2 (40.0)	0 (0.0)	2 (6.9)
**Total**	765 (6.6)	419 (12.0)	121 (16.4)	1,305 (8.3)	**Total**	301 (2.6)	163 (4.7)	51 (6.9)	515 (3.3)
**Any peripheral event**					**Ulcer/gangrene**				
**N**	**A1**	**A2**	**A3**	**Total**	**n**	**A1**	**A2**	**A3**	**Total**
**G1**	99 (2.7)	51 (5.5)	11 (9.2)	161 (3.5)	**G1**	53 (1.5)	38 (4.1)	9 (7.5)	100 (2.2)
**G2**	247 (4.0)	133 (8.1)	29 (11.9)	409 (5.0)	**G2**	130 (2.1)	80 (4.8)	23 (9.4)	233 (2.9)
**G3a**	102 (8.1)	68 (12.1)	25 (18.4)	195 (10.0)	**G3a**	53 (4.2)	42 (7.5)	17 (12.5)	112 (5.7)
**G3b**	23 (6.6)	48 (18.3)	24 (17.7)	95 (12.7)	**G3b**	16 (4.6)	33 (12.6)	13 (9.6)	62 (8.3)
**G4**	3 (5.0)	8 (9.8)	19 (21.8)	30 (13.1)	**G4**	2 (3.3)	6 (7.3)	13 (14.9)	21 (9.2)
**G5**	1 (11.1)	2 (40.0)	2 (13.3)	5 (17.2)	**G5**	1 (11.1)	2 (40.0)	1 (6.7)	4 (13.8)
**Total**	475 (4.1)	310 (8.9)	110 (14.9)	895 (5.7)	**Total**	255 (2.2)	201 (5.8)	76 (10.3)	532 (3.4)
**Non-advanced DR**					**Advanced DR**				
**N**	**A1**	**A2**	**A3**	**Total**	**n**	**A1**	**A2**	**A3**	**Total**
**G1**	389 (10.8)	131 (14.1)	16 (13.3)	536 (11.5)	**G1**	212 (5.9)	127 (13.6)	21 (17.5)	360 (7.7)
**G2**	657 (10.5)	258 (15.6)	37 (15.2)	952 (11.7)	**G2**	425 (6.8)	229 (13.9)	63 (25.8)	717 (8.8)
**G3a**	165 (13.2)	105 (18.7)	30 (22.1)	300 (15.4)	**G3a**	120 (9.6)	91 (16.2)	40 (29.4)	251 (12.9)
**G3b**	43 (12.3)	50 (19.0)	30 (22.1)	123 (16.4)	**G3b**	47 (13.4)	56 (21.3)	35 (25.7)	138 (18.4)
**G4**	10 (16.7)	12 (14.6)	18 (20.7)	40 (17.5)	**G4**	8 (13.3)	19 (23.2)	39 (44.8)	66 (28.8)
**G5**	0 (0.0)	1 (20.0)	5 (33.3)	6 (20.7)	**G5**	0 (0.0)	1 (20.0)	7 (46.7)	8 (27.6)
**Total**	1,264 (11.0)	557 (15.9)	136 (18.4)	1,957 (12.4)	**Total**	812 (7.0)	523 (15.0)	205 (27.8)	1,540 (9.8)

*CVD*, cardiovascular disease; *AMI*, acute myocardial infarction; *DR*, diabetic retinopathy; *eGFR*, estimated glomerular filtration rate.

eGFR (ml/min/1.73 m^2^): G1 = ≥90; G2 = 60-89; G3a = 45-59; G3b = 30-44; G4 = 15-29; G5 = <15; albuminuria: A1 = mormoalbuminuria; A2 = microalbuminuria; A3 = macroalbuminuria.

**Figure 2:**
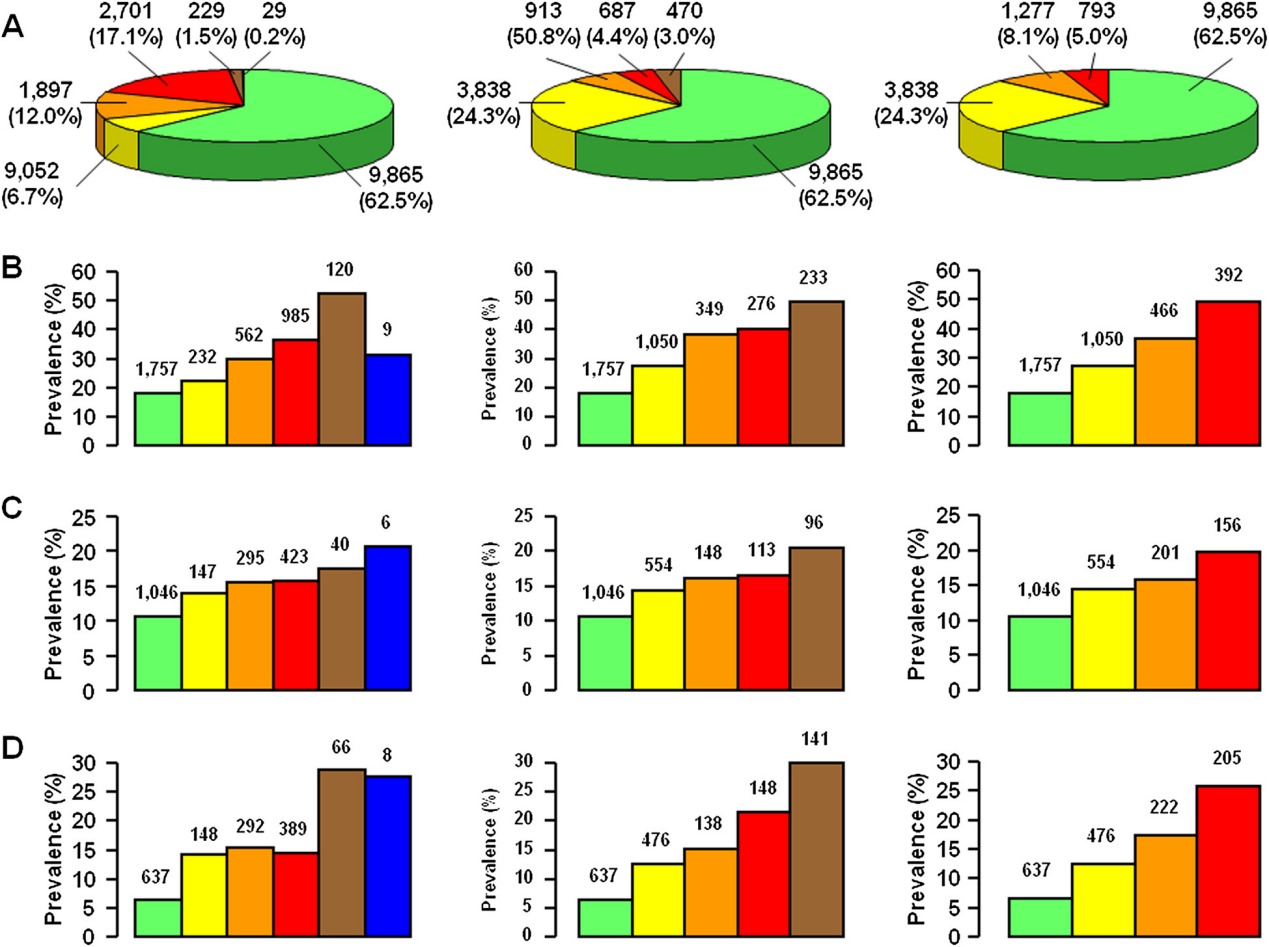
**Cases (% of total, A) and prevalence (% of cases, number of subjects on top of columns) of any CVD (B) and non-advanced (C) and advanced (D) DR according to CKD NFK’s KDOQI stage (left), AKDN risk category (middle), and KDIGO risk category (right).** NFK’s KDOQI classification: stage 0 (green), 1 (yellow), 2 (orange), 3 (red), 4 (brown), and 5 (blue); AKDN alternate system: risk category 0 (green), 1 (yellow), 2 (orange), 3 (red), and 4 (brown); KDIGO classification: risk category low (green), moderate (yellow), high (orange), and very high (red).

Irrespective of the classification system, 62.5% of patients had no CKD and 37.5% had various degrees of renal impairment, as previously detailed [8]: 18.8% had albuminuria alone, and 18.7% had reduced eGFR, either with (43.4%) or without (56.6%) albuminuria. However, while the percentages of subjects with stages 1-2 and stages 3-5 of the NKF’s KDOQI classification were almost identical, reclassification of nonalbuminuric patients assigned to stages 3a (n = 1,253) and 3b (n = 351) and microalbuminuric patients assigned to stage 2 (n = 1,653) and 3a (n = 562) into risk category 1 or moderate and risk category 2 or high with the AKDN and KDIGO systems, respectively, resulted in a decreasing number of subjects with increasing risk category, in keeping with the goal of reducing referral for specialist care. Moreover, since macroalbuminuric subjects with normal-to-high or mildly reduced eGFR (n = 120 and n = 244, respectively; NKF’s KDOQI stages 1-2) are classified into a higher risk category with the AKDN alternate system (i.e. risk category 3) than with the KDIGO classification (i.e. high), the number of patients decreased less strikingly from AKDN risk category 2 to 3 than from the KDIGO risk category high to very high (Figure 2).

**Figure 3:**
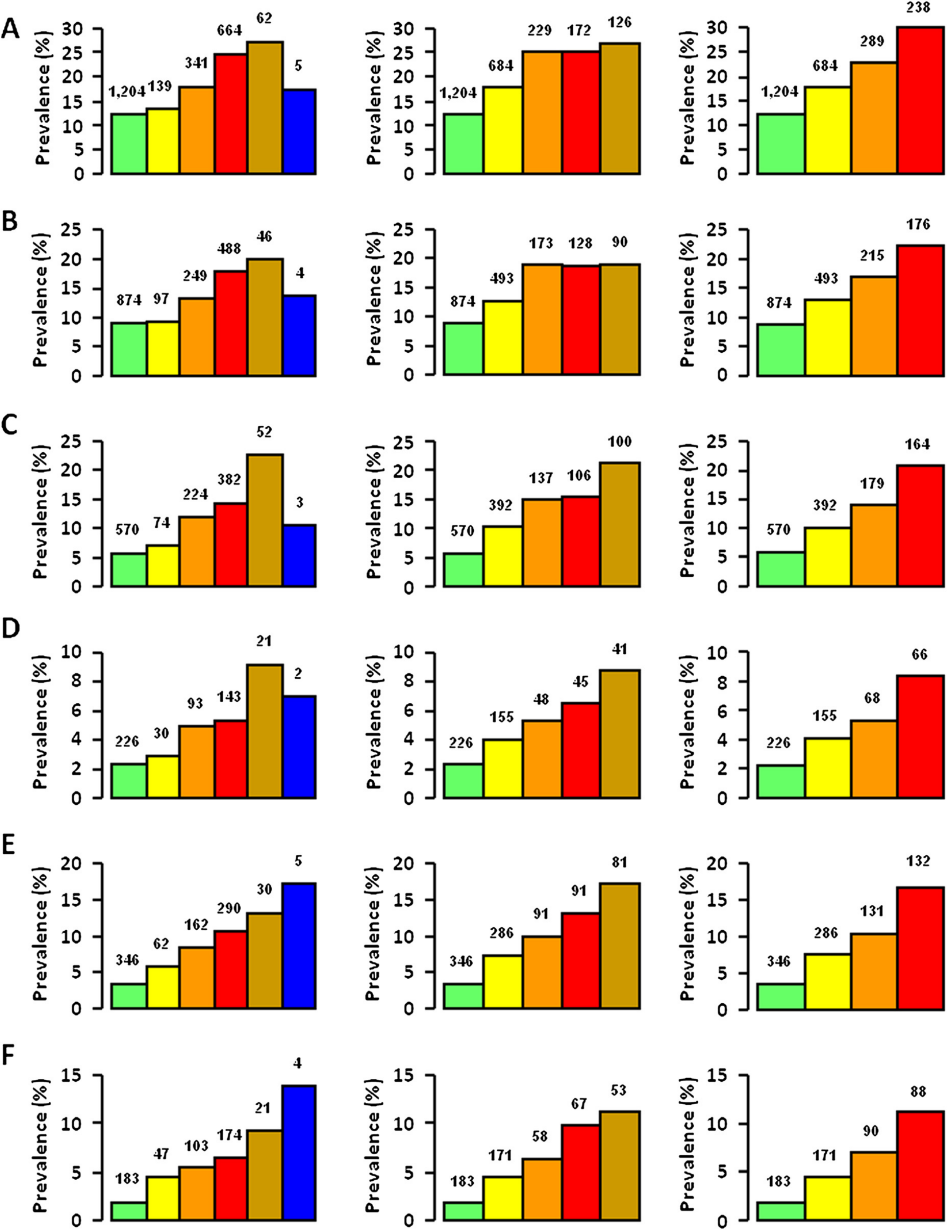
**Prevalence (% of cases, number of subjects on top of columns) of any coronary event (A), AMI (B), any cerebrovascular event (C), stroke (D), any peripheral event (E), and ulcer/gangrene (F) according to CKD NFK’s KDOQI stage (left), AKDN risk category (middle), and KDIGO risk category (right).** NFK’s KDOQI classification: stage 0 (green), 1 (yellow), 2 (orange), 3 (red), 4 (brown), and 5 (blue); AKDN alternate system: risk category 0 (green), 1 (yellow), 2 (orange), 3 (red), and 4 (brown); KDIGO classification: risk category low (green), moderate (yellow), high (orange), and very high (red).

**Figure 4:**
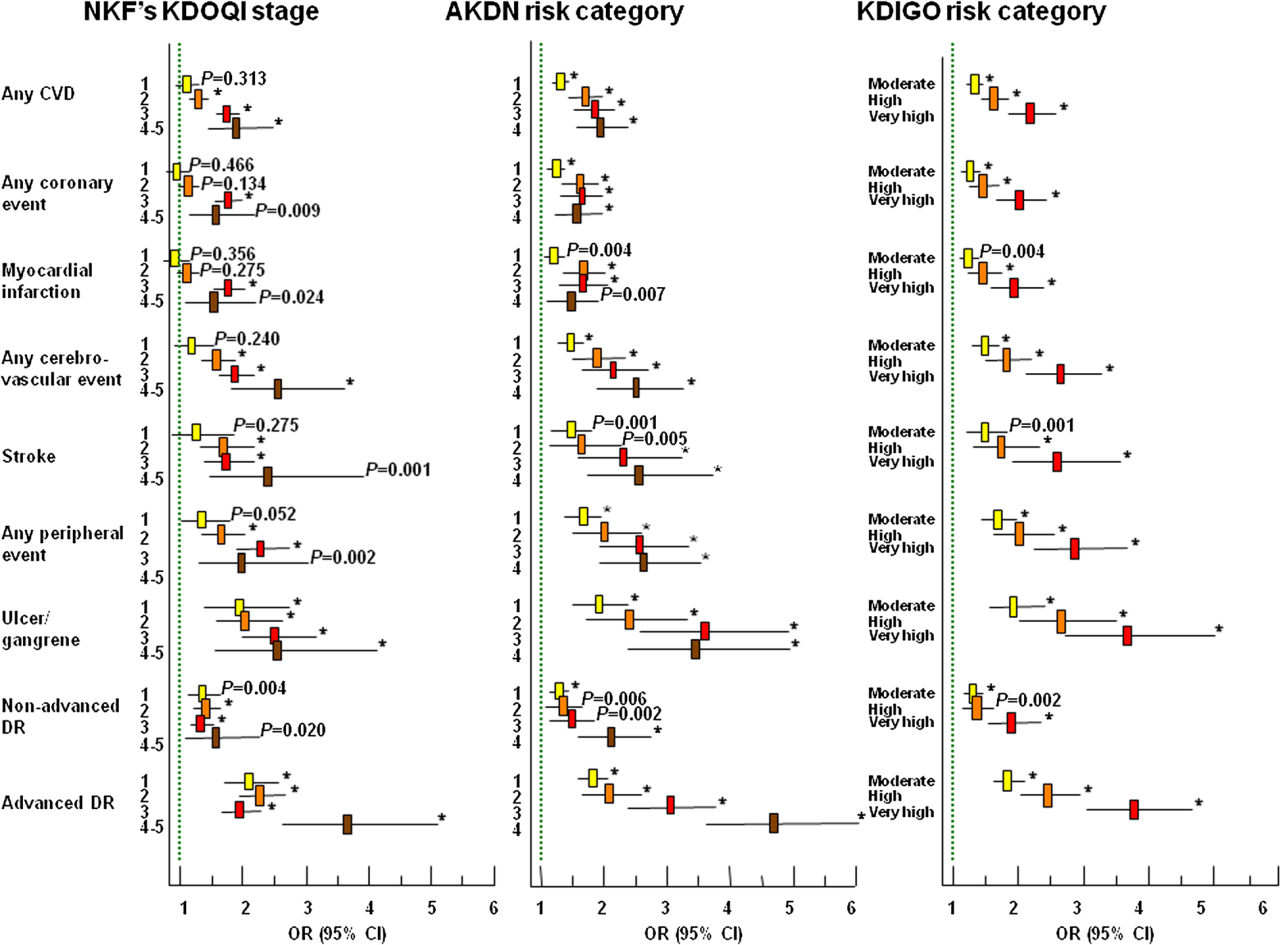
Independent* relation of any CVD, any coronary event, AMI, any cerebrovascular event, stroke, any peripheral event, ulcer/gangrene, and non-advanced and advanced DR with CKD NKF’s KDOQI stages, and AKDN and KDIGO risk categories. * Confounders: age, gender, smoking habits, diabetes duration, HbA_1c_, anti-hyperglycemic treatment, triglycerides, HDL cholesterol, dyslipidemia, and hypertension. The green lines indicate the reference categories (i.e. no CKD stage, risk category 0 and risk category low for NKF’s KDOQI, AKDN, and KDIGO classifications, respectively). * P<0.0001.

Though prevalence of complications increased with increasing CKD severity with all systems (*P* for trend <0.0001), it differed significantly between NKF’s KDOQI stages and AKDN or KDIGO risk categories (Figures 2 and 3). In fact, since prevalence of CVD, but not DR, was higher in patients with nonalbuminuric renal impairment (NKF’s KDOQI stages 3-5) than in micro and macroalbuminuric subjects with normal-to-high or mildly reduced eGFR (NKF’s KDOQI stages 1-2) (Table 1) [8,25], prevalence of any CVD, any coronary events, and myocardial infarction was significantly higher in risk category 1 or moderate than in stage 1 (*P* at least <0.001) as well as in risk category 2 (*P* < 0.0001) or high (*P* at least <0.005) than in stage 2, whereas prevalence of advanced DR was higher in risk category 3 or very high than in stage 3 (*P* < 0.0001). Moreover, since CVD prevalence was relatively low in macroalbuminuric subjects with non-reduced eGFR (included in AKDN and KDIGO risk category 3 and high, respectively) [8], it tended to plateau between AKDN risk categories 2 and 3, whereas they increased markedly from KDIGO risk category high to very high (Figures 2 and 3). However, a higher number of subjects without CVD (2,788) or DR (2,808) were appropriately classified in the lowest risk categories of the new systems (i.e. 1 and moderate, respectively) than in NKF’s KDOQI stage 1 (820 and 757, respectively). Logistic regression analysis showed that the strength of association of complications, independent of confounders, increased more progressively with AKDN and particularly KDIGO risk categories than with NKF’s KDOQI stages, except for cerebrovascular and peripheral events. In addition, NKF’s KDOQI stage 1 was not significantly associated with any CVD event and CVD events by vascular bed, except ulcer/gangrene, whereas stage 2 did not correlate with any CVD and coronary events. Finally, the odd ratios for CVD, except cerebrovascular events, tended to plateau between AKDN risk categories 3 and 4 or even 2 and 4 (Figure 4).

Likewise, significant differences were detected when prevalence of complications in eGFR and albuminuria categories grouped into the same CKD stage or risk category were compared. As previously reported [8], prevalence of CVD was higher in normoalbuminuric subjects with mildly reduced than in those with normal-to-high eGFR (no CKD with the NKF’s KDOQI classification, corresponding to risk categories 0 and low with the new systems, *P* = 0.03 to <0.0001) and that of CVD and DR in individuals with reduced eGFR and albuminuria than in those without albuminuria (NKF’s KDOQI CKD stages 3-5, *P* = 0.002 to <0.0001) (Table 1). Moreover, logistic regression analysis showed that, within AKDN risk category 1 and the corresponding KDIGO risk category moderate, the independent relation of CVD events, except stroke and ulcer/gangrene, was 1.5-to-2.0-fold higher with G3a-A1 (normoalbuminuric subjects with eGFR 45-59 ml/min/1.73 m^2^) and 1.2-to-1.7-fold higher with G2-A2 (microalbuminuric subjects with eGFR 60-89 ml/min/1.73 m^2^) than with G1-A2 (microalbuminuric subjects with eGFR ≥90 ml/min/1.73 m^2^) categories (Table 2), consistent with the finding that prevalence of CVD is higher in nonalbuminuric stages 3-5 than in stages 1-2 [8]. Conversely, the strength of correlation of advanced DR was lower with G3a-A1 than with G2-A2 and G1-A2 categories (Table 2), in keeping with the weaker association of this complication with reduced eGFR than with albuminuria [25]. Less striking were differences among eGFR and albuminuria categories grouped into AKDN risk categories 2 and 3 and KDIGO risk category high (not shown).

**Table 2:** Independent* relation of any CVD, any coronary event, AMI, any cerebrovascular event, stroke, any peripheral event, ulcer/gangrene, and non-advanced and advanced DR with eGFR and albuminuria categories grouped into AKDN risk category 1 and the corresponding KDIGO risk category moderate

**eGFR and albuminuria category**	**G1-A2**	**G2-A2**		**G3a-A1**	
**Complication**	**OR**	**OR (95 % CI)**	***P***	**OR (95 % CI)**	***P***
**Any CVD**	1.0	1.273 (1.038-1.562)	0.020	1.595 (1.264-2.012)	<0.0001
**Any coronary event**	1.0	1.293 (1.011 1.653)	0.040	1.883 (1.431 2.477)	<0.0001
**AMI**	1.0	1.294 (0.974-1.718)	0.075	1.907 (1.390-2.614)	<0.0001
**Any cerebrovascular event**	1.0	1.483 (1.080-2.036)	0.015	1.641 (1.147-2.349)	0.007
**Stroke**	1.0	1.708 (1.048-2.783)	0.032	1.587 (0.916-2.748)	0.099
**Any peripheral event**	1.0	1.409 (0.988 2.009)	0.058	2.005 (1.359 2.959)	<0.0001
**Ulcer/gangrene**	–	–	–	–	–
**Non-advanced DR**	1.0	0.994 (0.779-1.267)	0.961	0.771 (0.584 1.017)	0.065
**Advanced DR**	1.0	1.020 (0.784-1.328)	0.883	0.647 (0.474 0.884)	0.006

*Confounders: age, gender, smoking habits, diabetes duration, HbA_1c_, anti-hyperglycemic treatment, triglycerides, HDL cholesterol, dyslipidemia, and hypertension.

*CVD*, cardiovascular disease; *AMI*, acute myocardial infarction; *DR*, diabetic retinopathy; *eGFR*, estimated glomerular filtration rate; *KDIGO*, kidney disease: improving global outcomes; *OR*, odd ratio; *CI*, confidence interval.

G1-A2: eGFR ≥90 ml/min/1.73 m^2^ with microalbuminuria (reference category); G2-A2: eGFR 60-89 ml/min/1.73 m^2^ with microalbuminuria; G3a-A1: eGFR 45-59 ml/min/1.73 m^2^ with normoalbuminuria.

## Discussion

In this cross-sectional analysis of the RIACE study, we compared three CKD classification systems regarding their ability to define CVD and DR burden. As reported in the general population [18,20,28], the percentage of patients assigned to more advanced stages or risk categories decreased markedly when individuals staged with the NKF’ KDOQI classification (18.75% assigned to stage ≥3) were reclassified with the AKDN (7.34% assigned to risk category ≥3) and particularly the KDIGO (5.03% assigned to risk category very high) systems. This implies that, according to the rationale of the new classifications [18,20], a lower number of subjects with type 2 diabetes would be referred for specialist care, with significant cost savings. It remains to be clarified whether reclassification also improves accuracy of referral in terms of better prediction of morbidity and mortality in diabetic individuals.

Previous studies in the general population using the AKDN alternate system have shown that it better predicts adverse renal outcomes and all-cause mortality [18], whereas the NKF’ KDOQI classification is superior for identifying CKD complications such as anemia, acidosis, hyperphosphatemia, hyperparathyroidism and hypertension (but not hypoalbuminemia) [28]. However, reclassification with the alternate system was less accurate for mortality than for the renal outcome, since it consistently and incorrectly reclassified more patients who died to a lower category [18], whereas it more likely assigned to lower stages patients without each CKD complication [28], as compared the NKF’ KDOQI classification.

Our study focused on CVD and DR, the main complications of diabetes which affect more frequently diabetic patients with CKD than those without [25,29], and profoundly impact on life expectancy and quality of these individuals [30,31].

Results showed that prevalence of complications differed significantly between NKF’s KDOQI stages and AKDN or KDIGO risk categories and also among eGFR and albuminuria categories grouped into the same risk category. As expected, a large number of subjects without CVD and DR were correctly reclassified in the lowest risk category of the new systems. Moreover, the AKDN and particularly the KDIGO classification showed a more graded association with CVD and DR than the NKF’s KDOQI staging system, independently of confounders, thus supporting the concept that the new systems provide a better definition of CVD and DR burden in type 2 diabetes, consistent with previous data on renal outcomes and all-cause mortality from the general population [18]. Under the assumption that subjects assigned to the lowest risk category would not be referred for specialist care, at variance with those classified as AKDN risk categories ≥2 or KDIGO risk categories ≥ high, this would result in more accurate and less expensive management of diabetic individuals with CKD.

However, CVD, but not DR prevalence was higher in risk category 1 or moderate than in stage 1 (except ulcer/gangrene) as well as in risk category 2 or high than in stage 2, consistent with previous data comparing the AKDN alternate system with the NKF’s KDOQI classification in the NHANES cohort [28]. In addition, prevalence differed also among eGFR and albuminuria categories grouped into AKDN and KDIGO risk category 1 and moderate, respectively, and to a lesser extent into higher risk categories of the new systems. Both findings are attributable to the relatively high prevalence of CVD in individuals with nonalbuminuric renal impairment, higher than that in subjects with albuminuria and normal-to-high or mildly reduced eGFR [8], especially for coronary events [26]. This might imply that assignment of individuals with type 2 diabetes and normoalbuminuria who fall into eGFR category 3a and 3b to risk categories 1 (or moderate) and 2 (or high), respectively, should be tested in large prospective studies and eventually reconsidered with classification of these subjects into higher risk categories. Hopefully, the follow-up of the RIACE Study will provide insight into this issue.

Strengths of this study include the large size of the cohort, the completeness of data and the analysis of a contemporary dataset. The main limitation is the cross-sectional design of the study. Potential limitations concerning assessment of CKD and DR, including non-centralized measurements of albuminuria and serum creatinine and the use of funduscopy have been addressed in previous RIACE reports [8,21,25]. Briefly, as an external quality control of urinary albumin assays, 50 samples from each center were re-analyzed at the reference laboratory of the Coordinating center, confirming that the coefficient of variations between the peripheral and the central values were <15%, at least in the relevant clinical range of 15-500 mg/L [8,21]. Moreover, fundus was examined by an expert an ophthalmologist in each center, who was asked to fill in a standardized report format for classifying the RIACE participants [25].

In conclusions, the new AKDN and KDIGO systems seem to perform better than the NKF’s KDOQI classification in grading CVD and DR burden in subjects with type 2 diabetes. However, though these systems reclassify upward a large proportion of patients without complications, thus reducing unnecessary referrals, they appear to underestimate the burden from CVD, but not DR in diabetic subjects assigned to lower risk categories, especially those with reduced eGFR without albuminuria (see Additional file 1: Table S4). This suggests that the new systems should be tested for CVD and DR outcomes in prospective studies, in order to improve risk stratification of diabetic individuals with CKD.

## Competing interests

The authors declare that they have no competing interests.

## Authors’ contributions

All Authors have made substantial contributions to conception and design, or acquisition of data, or analysis and interpretation of data; have been involved in drafting the manuscript or revising it critically for important intellectual content; and have given final approval of the version to be published.

## Supplementary Material

Additional file 1: Table S1 Clinical characteristics of study subjects according to the NKF’s KDOQI CKD classification. **Table S2.** Clinical characteristics of study subjects according to the AKDN alternate CKD classification system. **Table S3.** Clinical characteristics of study subjects according to the KDIGO CKD classification. **Table S4.** Summary of the main study findings. Click here for file

## **The RIACE Study Group**

The RIACE Steering Committee

Giuseppe Pugliese (Coordinator), Giuseppe Penno (Secretariat), Anna Solini, Enzo Bonora, Emanuela Orsi, Roberto Trevisan, Luigi Laviola, Antonio Nicolucci.

The Diabetic Nephropathy Study Group, SID

Giuseppe Pugliese, Salvatore De Cosmo, Gabriella Gruden, Susanna Morano, Giuseppe Penno, Francesco Pugliese, Giampaolo Zerbini, Luigi Laviola, Anna Solini, Roberto Trevisan.

Participating diabetes centres

1. Azienda Ospedaliera Sant'Andrea, Roma (Coordinating Center): Giuseppe Pugliese, Laura Salvi, Lucilla Bollanti and Alessandra Bazuro.

2. Ospedale Le Molinette, Torino: Paolo Cavallo-Perin, Gabriella Gruden and Bartolomeo Lorenzati.

3. Ospedale San Luigi Gonzaga, Orbassano: Mariella Trovati, Giovanni Anfossi†, Franco Cavalot and Massimo Chirio.

4. Ospedale San Raffaele, Milan: Giampaolo Zerbini, Valentina Martina and Silvia Maestroni.

5. IRCCS “Cà Granda – Ospedale Maggiore Policlinico”, Milan: Emanuela Orsi, Laura Montefusco and Dario Zimbalatti.

6. Ospedale San Paolo, Milan: Antonio Pontiroli, Annamaria Veronelli and Barbara Zecchini.

7. Ospedale San Giuseppe, Milan: Maura Arosio and Alessia Dolci.

8. Ospedali Riuniti, Bergamo: Roberto Trevisan and Anna Corsi.

9. Ospedale Maggiore, Verona: Enzo Bonora and Giacomo Zoppini.

10. Policlinico Universitario, Padova: Angelo Avogaro, Monica Vedovato and Elisa Pagnin.

11. Ospedale Cisanello, Azienda Ospedaliero-Universitaria Pisana, Pisa: Giuseppe Penno, Laura Pucci, Daniela Lucchesi, Eleonora Russo and Monia Garofolo.

12. Ospedale Santa Chiara, Azienda Ospedaliero-Universitaria Pisana, Pisa: Anna Solini.

13. Ospedale Le Scotte, Siena: Francesco Dotta, Cecilia Fondelli and Laura Nigi.

14. Policlinico Umberto I, Roma: Susanna Morano and Alessandra Gatti.

15. Ospedale S. Maria Goretti, Latina: Raffaella Buzzetti and Chiara Foffi.

16. Ospedali Riuniti, Foggia: Mauro Cignarelli, Olga Lamacchia, Sabina Pinnelli and Lucia Monaco.

17. Policlinico Universitario, Bari: Francesco Giorgino, Luigi Laviola and Sebastio Perrini.

18. Policlinico Mater Domini, Catanzaro: Giorgio Sesti and Francesco Andreozzi.

19. Policlinico Monserrato, Cagliari: Marco Giorgio Baroni and Giuseppina Frau.

^^^ Dr Anfossi is deceased.
