# Supplementary material for: Distribution of cardiovascular disease and retinopathy in patients with type 2 diabetes according to different classification systems for chronic kidney disease: a cross-sectional analysis of the renal insufficiency and cardiovascular events (RIACE) Italian multicenter study
Source: Cardiovasc Diabetol. 2014 Mar 13;13:59. doi: 10.1186/1475-2840-13-59 (PMC4008155; doi:10.1186/1475-2840-13-59)
Supplement: Additional file 1: Table S1 — Clinical characteristics of study subjects according to the NKF’s KDOQI CKD classification. Table S2. Clinical characteristics of study subjects according to the AKDN alternate CKD classification system. Table S3. Clinical characteristics of study subjects according to the KDIGO CKD classification. Table S4. Summary of the main study findings. [file 1475-2840-13-59-S1.doc]

**Table S1.** Clinical characteristics of study subjects according to the NKF’s KDOQI CKD classification.

| **Variables** | **CKD NFK’s KDOQI stages** | | | | | ***P*** |
| --- | --- | --- | --- | --- | --- | --- |
|  | **0** | **1** | **2** | **3** | **4-5** |  |
| **N (% of total)** | 9,865 (62.5) | 1,052 (6.7) | 1,897 (12.0) | 2,701 (17.1) | 258 (1.6) |  |
| **Age, years** | 64.3±10.1 | 61.6±10.4 | 68.0±9.5 | 72.2±8.8 | 73.2±8.9 | 0.0001 |
| **Males, n (%)** | 5,526 (56.0) | 764 (72.6) | 1,321 (69.6) | 1,226 (45.4) | 123 (47.7) | 0.0001 |
| **Smoking, n (%)** |  |  |  |  |  | 0.0001 |
| **Never** | 5,695 (57.7) | 483 (45.9) | 961 (50.7) | 1,642 (60.8) | 147 (57.0) |  |
| **Former** | 2,652 (26.9) | 301 (28.6) | 621 (32.7) | 786 (29.1) | 74 (28.7) |  |
| **Current** | 1,518 (15.4) | 268 (25.5) | 315 (16.6) | 273 (10.1) | 37 (14.3) |  |
| **Age at diabetes diagnosis (years)** | 52.4±11.1 | 49.6±11.0 | 53.0±11.3 | 55.7±11.8 | 53.3±12.8 | 0.0001 |
| **Diabetes duration (years)** | 11.9±9.7 | 12.0±9.2 | 15.0±10.3 | 16.5±11.0 | 19.9±11.2 | 0.0001 |
| **HbA_1c_ (%)** | 7.40±1.41 | 7.97±1.75 | 7.83±1.63 | 7.72±1.59 | 7.73±1.65 | 0.0001 |
| **Anti-hyperglicemic treatment, n (%)** |  |  |  |  |  | 0.0001 |
| **Diet** | 1,559 (15.8) | 90 (8.6) | 190 (10.0) | 264 (9.8) | 23 (8.9) |  |
| **OHA** | 6,325 (64.1) | 670 (63.7) | 1,127 (59.4) | 1,477 (54.7) | 82 (31.8) |  |
| **OHA + insulin** | 817 (8.3) | 141 (13.4) | 264 (13.9) | 277 (10.3) | 20 (7.8) |  |
| **Insulin** | 1,164 (11.8) | 151 (14.4) | 316 (16.7) | 683 (25.3) | 133 (51.6) |  |
| **Triglycerides (mmol/l)** | 1.47±0.89 | 1.74±1.50 | 1.69±1.04 | 1.74±1.02 | 2.08±1.27 | 0.0001 |
| **Total cholesterol (mmol/l)** | 4.78±0.96 | 4.76±1.05 | 4.76±1.01 | 4.81±1.05 | 4.90±1.14 | 0.161 |
| **HDL cholesterol (mmol/l)** | 1.32±0.35 | 1.23±0.35 | 1.25±0.34 | 1.26±0.36 | 1.19±0.42 | 0.0001 |
| **LDL cholesterol (mmol/l)** | 2.80±0.83 | 2.77±0.87 | 2.75±0.85 | 2.77±0.88 | 2.76±0.87 | 0.033 |
| **Non-HDL cholesterol (mmol/l)** | 3.47±0.92 | 3.53±1.03 | 3.50±0.98 | 3.55±0.99 | 3.70±1.06 | 0.0001 |
| **Dyslipidemia, n (%)** | 8,082 (81.9) | 843 (80.1) | 1,553 (81.9) | 2,265 (83.9) | 217 (84.1) | 0.052 |
| **Lipid-lowering treatment, n (%)** | 4,310 (43.7) | 447 (42.5) | 916 (48.3) | 1,473 (54.5) | 140 (54.3) | 0.0001 |
| **SBP (mmHg)** | 136.9±17.4 | 139.1±18.7 | 140.9±18.9 | 139. 5±18.8 | 142.0±21.0 | 0.0001 |
| **DBP (mmHg)** | 78.8±9.2 | 80.0±9. 8 | 79.4±9.8 | 77. 8±9.7 | 77.7±11.0 | 0.0001 |
| **Hypertension, n (%)** | 7,791 (79.0) | 902 (85.7) | 1,727 (91.0) | 2,522 (93.4) | 247 (95.7) | 0.0001 |
| **Anti-hypertensive treatment, n (%)** | 6,241 (63.3) | 770 (73.2) | 1,547 (81.5) | 2,348 (86.9) | 244 (94.6) | 0.0001 |
| **RAS blockers, n (%)** | 5,016 (50.8) | 670 (63.7) | 1,328 (70.0) | 1,955 (72.4) | 196 (76.0) | 0.0001 |
| **BMI (kg/m^2^)** | 28.70±5.09 | 29.72±5.76 | 29.43±4.93 | 29.21±5.07 | 30.15±5.94 | 0.0001 |
| **Waist circumference (cm)** | 101.8±11.0 | 104.5±12.3 | 103.8±10.7 | 102.9±10.8 | 104.8±12.7 | 0.0001 |
| **Albuminuria (mg /24 h)** | 10.7±7.1 | 175.9±639.8 | 175.2±424.1 | 136.6±383.6 | 578.5±1017.5 | 0.0001 |
| **Serum creatinine (μmol/l)** | 75.1±15.0 | 63.6±10.6 | 84.9±12.4 | 114.9±23.9 | 242.2±120.2 | 0.0001 |
| **eGFR (ml/min/1.73 m^2^)** | 86.86±18.65 | 109.06±19.00 | 74.91±8.36 | 49.50±7.88 | 23.14±6.09 | 0.0001 |

Data are mean±SD or number of subjects (%). *P* values for comparison among groups using the one-way ANOVA and the Kruskall-Wallis for parametric and non-parametric continuous variables, respectively, and the Pearson χ^2^ for categorical variables. The 29 subjects falling into the NKF’s KDOQI stage (and eGFR category) 5 were grouped with individuals assigned to NKF’s KDOQI stage (and eGFR category) 4. NKF’s KDOQI = National Kidney Foundation’s Kidney Disease Outcomes Quality Initiative; CKD = chronic kidney disease; HbA_1c_ = hemoglobin A_1c_; OHA = oral hypoglycemic agent; SBP = systolic blood pressure; DBP = diastolic blood pressure; RAS = renin-angiotensin system; BMI = doby mass index; eGFR = estimated glomerular filtration rate.

**Table S2.** Clinical characteristics of study subjects according to the AKDN alternate CKD classification system.

| **Variables** | **AKDN risk categories** | | | | | ***P*** |
| --- | --- | --- | --- | --- | --- | --- |
|  | **0** | ***P*** | **2** | **3** | **4** |  |
| **N (% of total)** | 9,865 (62.5) | 3,838 (24.3) | 913 (5.8) | 687 (4.4) | 470 (3.0) | 0.0001 |
| **Age, years** | 64.3±10.1 | 67.9±10.2 | 72.8±8.5 | 68.7±10.6 | 71.2±9.6 |  |
| **Males, n (%)** | 5,526 (56.0) | 2,247 (58.5) | 473 (51.8) | 421 (61.3) | 293 (62.3) | 0.0001 |
| **Smoking, n (%)** |  |  |  |  |  | 0.0001 |
| **Never** | 5,695 (57.7) | 2,124 (55.3) | 531 (58.2) | 335 (48.8) | 243 (51.7) | 0.0001 |
| **Former** | 2,652 (26.9) | 1,104 (28.8) | 291 (31.9) | 235 (34.2) | 152 (32.3) | 0.0001 |
| **Current** | 1,518 (15.4) | 610 (15.9) | 91 (10.0) | 117 (17.0) | 75 (16.0) | 0.0001 |
| **Age at diabetes diagnosis (years)** | 52.4±11.1 | 53.5±11.6 | 55.7±11.4 | 52.5±12.3 | 52.4±12.3 | 0.0001 |
| **Diabetes duration (years)** | 11.9±9.7 | 14.3±10.4 | 17.1±10.9 | 16.2±10.8 | 18.8±10.9 | 0.0001 |
| **HbA_1c_ (%)** | 7.40±1.41 | 7.76±1.58 | 7.75±1.62 | 8.02±1.91 | 7.89±1.61 | 0.0001 |
| **Anti-hyperglicemic treatment, n (%)** |  |  |  |  |  | 0.0001 |
| **Diet** | 1,559 (15.8) | 413 (10.8) | 82 (9.0) | 43 (6.3) | 29 (6.2) |  |
| **OHA** | 6,325 (64.1) | 2,333 (60.8) | 488 (53.5) | 354 (51.5) | 181 (38.5) |  |
| **OHA + insulin** | 817 (8.3) | 462 (12.0) | 94 (10.3) | 95 (13.8) | 51 (10.9) |  |
| **Insulin** | 1,164 (11.8) | 630 (16.4) | 249 (27.3) | 195 (28.4) | 209 (44.5) |  |
| **Triglycerides (mmol/l)** | 1.47±0.89 | 1.74±1.50 | 1.69±1.04 | 1.74±1.02 | 2.08±1.27 | 0.0001 |
| **Total cholesterol (mmol/l)** | 4.78±0.96 | 4.76±1.05 | 4.76±1.01 | 4.81±1.05 | 4.90±1.14 | 0.001 |
| **HDL cholesterol (mmol/l)** | 1.32±0.35 | 1.23±0.35 | 1.25±0.34 | 1.26±0.36 | 1.19±0.42 | 0.0001 |
| **LDL cholesterol (mmol/l)** | 2.80±0.83 | 2.77±0.87 | 2.75±0.85 | 2.77±0.88 | 2.76±0.87 | 0.002 |
| **Non-HDL cholesterol (mmol/l)** | 3.47±0.92 | 3.53±1.03 | 3.50±0.98 | 3.55±0.99 | 3.70±1.06 | 0.0001 |
| **Dyslipidemia, n (%)** | 8,082 (81.9) | 843 (80.1) | 1,553 (81.9) | 2,265 (83.9) | 217 (84.1) | 0.060 |
| **Lipid-lowering treatment, n (%)** | 4,310 (43.7) | 447 (42.5) | 916 (48.3) | 1,473 (54.5) | 140 (54.3) | 0.0001 |
| **SBP (mmHg)** | 136.9±17.4 | 139.1±18.7 | 140.9±18.9 | 139. 5±18.8 | 142.0±21.0 | 0.0001 |
| **DBP (mmHg)** | 78.8±9.2 | 80.0±9. 8 | 79.4±9.8 | 77. 8±9.7 | 77.7±11.0 | 0.0001 |
| **Hypertension, n (%)** | 7,791 (79.0) | 902 (85.7) | 1,727 (91.0) | 2,522 (93.4) | 247 (95.7) | 0.0001 |
| **Anti-hypertensive treatment, n (%)** | 6,241 (63.3) | 770 (73.2) | 1,547 (81.5) | 2,348 (86.9) | 244 (94.6) | 0.0001 |
| **RAS blockers, n (%)** | 5,016 (50.8) | 670 (63.7) | 1,328 (70.0) | 1,955 (72.4) | 196 (76.0) | 0.0001 |
| **BMI (kg/m^2^)** | 28.70±5.09 | 29.72±5.76 | 29.43±4.93 | 29.21±5.07 | 30.15±5.94 | 0.0001 |
| **Waist circumference (cm)** | 101.8±11.0 | 104.5±12.3 | 103.8±10.7 | 102.9±10.8 | 104.8±12.7 | 0.0001 |
| **Albuminuria (mg /24 h)** | 10.7±7.1 | 175.9±639.8 | 175.2±424.1 | 136.6±383.6 | 578.5±1017.5 | 0.0001 |
| **Serum creatinine (μmol/l)** | 75.1±15.0 | 63.6±10.6 | 84.9±12.4 | 114.9±23.9 | 242.2±120.2 | 0.0001 |
| **eGFR (ml/min/1.73 m^2^)** | 86.86±18.65 | 76.52±23.58 | 47.84±7.96 | 61.71±28.30 | 35.65±13.79 | 0.0001 |

Data are mean±SD or number of subjects (%). *P* values for comparison among groups using the one-way ANOVA and the Kruskall-Wallis for parametric and non-parametric continuous variables, respectively, and the Pearson χ^2^ for categorical variables. CKD = chronic kidney disease; HbA_1c_ = hemoglobin A_1c_; OHA = oral hypoglycemic agent; SBP = systolic blood pressure; DBP = diastolic blood pressure; RAS = renin-angiotensin system; BMI = doby mass index; eGFR = estimated glomerular filtration rate.

**Table S3.** Clinical characteristics of study subjects according to the KDIGO CKD classification.

| **Variables** | **KDIGO risk categories** | | | | ***P*** |
| --- | --- | --- | --- | --- | --- |
|  | **Low** | **Moderate** | **High** | **Very high** |  |
| N (% of total) | 9,865 (62.5) | 3,838 (24.3) | 1,277 (8.1) | 793 (5.0) |  |
| Age, years | 64.3±10.1 | 67.9±10.2 | 70.6±9.6 | 71.9±9.7 | 0.0001 |
| Males, n (%) | 5,526 (56.0) | 2,247 (58.5) | 747 (58.5) | 440 (55.5) | 0.026 |
| Smoking, n (%) |  |  |  |  | 0.0001 |
| Never | 5,695 (57.7) | 2,124 (55.3) | 679 (53.2) | 430 (54.2) |  |
| Former | 2,652 (26.9) | 1,104 (28.8) | 422 (33.0) | 256 (32.3) |  |
| Current | 1,518 (15.4) | 610 (15.9) | 176 (13.8) | 107 (13.5) |  |
| Age at diabetes diagnosis (years) | 52.4±11.1 | 53.5±11.6 | 54.1±11.6 | 53.5±12.6 | 0.0001 |
| Diabetes duration (years) | 11.9±9.7 | 14.3±10.4 | 16.4±10.8 | 18.4±11.0 | 0.0001 |
| HbA_1c_ (%) | 7.40±1.41 | 7.76±1.58 | 7.86±1.72 | 7.89±1.73 | 0.0001 |
| Anti-hyperglicemic treatment, n (%) |  |  |  |  | 0.0001 |
| Diet | 1,559 (15.8) | 413 (10.8) | 100 (7.8) | 54 (6.8) |  |
| OHA | 6,325 (64.1) | 2333 (60.8) | 699 (54.7) | 324 (40.9) |  |
| OHA + insulin | 817 (8.3) | 462 (12.0) | 161 (12.6) | 79 (10.0) |  |
| Insulin | 1,164 (11.8) | 630 (16.4) | 317 (24.8) | 336 (42.4) |  |
| Triglycerides (mmol/l) | 1.47±0.89 | 1.74±1.50 | 1.69±1.04 | 1.74±1.02 | 0.0001 |
| Total cholesterol (mmol/l) | 4.78±0.96 | 4.76±1.05 | 4.76±1.01 | 4.81±1.05 | 0.054 |
| HDL cholesterol (mmol/l) | 1.32±0.35 | 1.23±0.35 | 1.25±0.34 | 1.26±0.36 | 0.0001 |
| LDL cholesterol (mmol/l) | 2.80±0.83 | 2.77±0.87 | 2.75±0.85 | 2.77±0.88 | 0.009 |
| Non-HDL cholesterol (mmol/l) | 3.47±0.92 | 3.53±1.03 | 3.50±0.98 | 3.55±0.99 | 0.0001 |
| Dyslipidemia, n (%) | 8,082 (81.9) | 3,161 (82.4) | 744 (81.5) | 563 (82.0) | 0.375 |
| Lipid-lowering treatment, n (%) | 4,310 (43.7) | 1,862 (48.5) | 455 (49.8) | 381 (55.5) | 0.0001 |
| SBP (mmHg) | 139.9±17.4 | 139.4±18.4 | 139.3±19.4 | 141.1±19.5 | 0.0001 |
| DBP (mmHg) | 78.8±9.2 | 79.0±9.6 | 77.2±9.8 | 78.8±10.4 | 0.003 |
| Hypertension, n (%) | 7,791 (79.0) | 3,433 (89.4) | 856 (93.8) | 659 (95.9) | 0.0001 |
| Anti-hypertensive treatment, n (%) | 6,241 (63.3) | 3,041 (79.2) | 816 (89.4) | 621 (90.4) | 0.0001 |
| RAS blockers, n (%) | 5,016 (50.8) | 2,554 (66.5) | 691 (75.7) | 542 (78.9) | 0.0001 |
| BMI (kg/m^2^) | 28.70±5.09 | 29.36±5.17 | 29.06±5.10 | 29.85±5.45 | 0.0001 |
| Waist circumference (cm) | 101.8±11.0 | 103.4±11.0 | 102.8±11.1 | 104.7±11.9 | 0.0001 |
| Albuminuria (mg /24 h) | 10.7±7.1 | 59. 8±59.5 | 65.6±68.5 | 480.9±995.8 | 0.0001 |
| Serum creatinine (μmol/l) | 75.1±15.0 | 84.9±19.4 | 119.3±21.2 | 114.0±43.3 | 0.0001 |
| eGFR (ml/min/1.73 m^2^) | 86.86±18.65 | 76.52±23.58 | 58.25±20.64 | 35.86±11.38 | 0.0001 |

Data are mean±SD or number of subjects (%). *P* values for comparison among groups using the one-way ANOVA and the Kruskall-Wallis for parametric and non-parametric continuous variables, respectively, and the Pearson χ^2^ for categorical variables. KDIGO = Kidney Disease: Improving Global Outcomes; CKD = chronic kidney disease; HbA_1c_ = hemoglobin A_1c_; OHA = oral hypoglycemic agent; SBP = systolic blood pressure; DBP = diastolic blood pressure; RAS = renin-angiotensin system; BMI = doby mass index; eGFR = estimated glomerular filtration rate.

**Table S4.** Summary of the main study findings.

- Progressive increase of prevalence of CVD and DR with eGFR categories (except coronary and cerebrovascular events in G4-G5 and G5, respectively) and from normo to macroalbuminuria.
- Reclassification of a large number of subjects from NKF’s KDOQI stages 3a and 3b into lower AKDN and KDIGO risk categories, resulting into a decreasing number of patients with increasing risk category of the new systems.
- Increased prevalence of complications with increasing CKD severity with all three classification systems, but more progressive increase of strength of independent association of complications with AKDN and particularly KDIGO risk categories than with NKF’s KDOQI stages, except for cerebrovascular and peripheral events.
- Higher number of subjects without CVD or DR appropriately classified in the lowest risk categories of the new systems (i.e. 1 and moderate, respectively) than in NKF’s KDOQI stage 1.
- Higher number of subjects with CVD (especially any CVD, any coronary events, and myocardial infarction), but not DR, inappropriately classified in the lowest risk categories of the new systems (i.e. 1 and moderate, respectively) than in NKF’s KDOQI stage 1.
- Significant differences in the prevalence of complications among eGFR and albuminuria categories grouped into the same CKD stage (e.g. no CKD or nonalbuminuric vs. albuminuric stage 3) or risk category (e.g. within AKDN risk category 1 and the corresponding KDIGO risk category moderate and, to a lesser extent, within AKDN risk categories 2 and 3 and KDIGO risk category high.
